# Think Well Rather Than Evil

**Published:** 1880-10

**Authors:** 


### THINK WELL, RATHER THAN EVIL

I BELIEVE it to be a much better piece of wisdom to think the best of men rather than the worst. I had rather be cheated, once in a while, and hold to the general tenor of this trust, than to wear a double magnifying lens of suspicion, and be always safe. Nay, am I not cheated in this way just as much, and more? By adopting this suspicious method, I both cheat and am cheated. I cheat many an honest man of his just claim upon my regard and confidence, and I am cheated out of the blessedness of whole-hearted love and kindly association. Therefore the unmerciful man is most certainly an unblessed man. His sympathies are all dried up; he is afflicted with a chronic jaundice, and lives, timidly and darkly, in a little narrow rat-hole of distrust. He has no free use of the world; he breathes no liberal and generous air; he walks in no genial sunshine. He loses all the bliss that comes from sympathy, from open-heartedness, from familiar and confiding association. More than this, such a theory of humanity is an open self-condemnation. Whence has he derived this theory? Upon what premises has he built it up? Surely, from his own self-consciousness, from his own personal experience. There is darkness within him, and so darkness falls upon everything. His own actions are sinister, and so all humanity squints. The suspicious man, the man who distrusts all other men, and so is unmerciful to all, reveals himself as a mean man. For I urge, that not only is this an unmerciful view of men in general—it is an unjust view. The goodness of people around us is not all a mask. There is much that is "sounding brass and tinkling cymbal," but also there is sweet and true music. I believe those men who seem to us the worst, seem worse than they really are. I believe there is some vein of light in the darkest heart—some extenuating influence in the basest life. Now, it is well not to run into extremes, but to regard men as they are—creatures with mixed motives and complex natures. But if an extreme we must have—if we will adopt a sweeping theory respecting mankind in general—I repeat, it is better to think the best of them rather than the worst, and run the risk

